# Factors associated with tuberculosis deaths during hospitalization in Midwest Brazil

**DOI:** 10.1590/S1678-9946202567011

**Published:** 2025-02-17

**Authors:** Ademar Rodrigues de Oliveira, Cláudia Elizabeth Volpe-Chaves, Mara Luci Gonçalves Galiz Lacerda, Alexandre Albuquerque Bertucci, Bruna Abdul Ahad Saad, Caroline Tieppo Flores de Oliveira, James Venturini, Sandra Maria do Valle Leone de Oliveira, Anamaria Mello Miranda Paniago

**Affiliations:** 1 Hospital Universitário Maria Aparecida Pedrossian, Campo Grande, Mato Grosso do Sul, Brazil; 2 Hospital Regional de Mato Grosso do Sul, Campo Grande, Mato Grosso do Sul, Brazil; 3 Universidade Federal de Mato Grosso do Sul, Campo Grande, Mato Grosso do Sul, Brazil; 4 Fundação Oswaldo Cruz, Campo Grande, Mato Grosso do Sul, Brazil

**Keywords:** Pulmonary tuberculosis, Extrapulmonary tuberculosis, Disseminated tuberculosis, Mortality, Mycobacterium tuberculosis

## Abstract

Tuberculosis (TB) is a treatable disease and one of the leading causes of death worldwide, notably affecting people living with the human immunodeficiency virus. The COVID-2019 pandemic worsened TB outcomes, particularly in high-burden countries such as Brazil. Accurate data on the mortality of hospitalized patients is limited. This study aimed to evaluate clinical and epidemiological characteristics and identify mortality risk factors among all hospitalized patients with TB at a tertiary hospital in Midwest Brazil from 2017 to 2019. The median age of the 154 patients included in the study was 48 years (interquartile range: 38–59 years), and the majority were male (74.68%). The main comorbidities were diabetes mellitus and chronic obstructive pulmonary disease; 44 patients (28.57%) were admitted to the intensive care unit (ICU). The mortality rate was 33.12%, and the leading cause of death was septic shock, followed by respiratory failure due to TB. The main factors associated with death were age (*p*=0.001), central nervous system TB (*p*=0.026), reduced consciousness (*p*<0.001), ICU admission (*p*<0.001), mechanical ventilation (*p*<0.001), use of vasoactive drugs (VAD) (*p*<0.001), and duration of VAD use (*p*=0.038). A high rate of inpatient deaths was observed, reflecting the severity of TB and the challenges in its clinical management. It is imperative to implement strategies to reduce the mortality rate.

## INTRODUCTION

Tuberculosis (TB) is a global health problem associated with significant risks of disability and premature death, and is considered a reemerging disease. People living with the human immunodeficiency virus (PLHIV) are more likely to develop TB. Regardless of the presence of an HIV infection, timely diagnosis and treatment can cure TB in most cases^1-3^.

In 2019, TB was ranked as the 13^th^ leading cause of death worldwide, being the leading cause among infectious diseases. In 2022, the number of new TB diagnoses reached 7.5 million, marking a significant recovery in the number of people diagnosed and treated for TB after two years of COVID-19-related disruptions. TB remained the second leading cause of death from a single infectious agent, second only to COVID-19. Approximately 472,500 (6.3%) of these cases occurred in PLHIV, which has been declining for several years. India, Indonesia, China, the Philippines, Pakistan, Nigeria, Bangladesh, and the Democratic Republic of the Congo account for two-thirds of all TB cases worldwide, whereas only 2.2% of active TB cases have been reported in Europe^4,5^.

The TB incidence coefficient and the proportion of tested cases of PLHIV in Brazil increased significantly until 2019; however, in 2020, the COVID-19 pandemic caused a major reduction in the number of newly diagnosed TB cases and a consequent increase of TB-related deaths globally, regionally, and nationally^5^. Data from 2022 show a significant recovery in the number of TB cases diagnosed and treated two years after the pandemic. In 2023, the number of patients with TB slightly decreased, although these are preliminary data. Among PLHIV with TB, the incidence rate in Brazil increased from 8.6% in 2022 to 9.3% in 2023^6^.

Many studies have examined TB mortality rates in low-income developing countries and regions with high HIV prevalence^7,8^. However, data on mortality among hospitalized patients with TB remain sparse. Death certificate reports are often inaccurate, and research methodologies have increased the unreliability of existing data^9^. Research on the mortality rates of hospitalized patients with TB infection is essential to accurately identify predictors and quantify mortality, which could help develop effective control measures and reduce such rates^10^.

Given the critical need to establish strategies to reduce TB-related mortality, this study aimed to evaluate clinical and epidemiological characteristics of patients admitted to a tertiary hospital and to assess risk factors associated with mortality during hospitalization.

## MATERIALS AND METHODS

The study was conducted at the Hospital Regional de Mato Grosso do Sul (HRMS) from 2017 to 2019, and included all adult patients with confirmed or clinically diagnosed TB admitted during this period.

TB was confirmed by the presence of characteristic clinical manifestations of the disease, as well as the following criteria: a) presence of acid-fast bacillus (AFB) in a smear of biological material; b) presence of AFB in the histopathological examination; c) detection of *Mycobacterium tuberculosis* (*M. tb*) genetic material by the rapid molecular test on the GeneXpert^®^ platform; and d) identification of *M. tb* from the culture of a clinical sample in a medium specific for AFB.

Clinically diagnosed TB was defined based on clinical manifestations, compatible imaging tests, or histopathological examination findings compatible with TB but without identification of the pathogen.

The Hospital Epidemiology Nucleus Notification Database was searched for cases. The data collected included age, sex, profession, place of origin, ethnicity, duration of symptoms, and clinical form of TB. This information was gathered from electronic medical records using a data collection form. Laboratory test results, histopathological data, and imaging results used to define the case were also extracted from electronic medical records.

A reduction in the level of consciousness is considered any alteration in the Glasgow Coma Scale scores for eye opening or verbal response at admission^11^.

### Statistical analysis

Categorical variables were compared using the chi-squared test or Fisher’s exact test. Numerical variables were compared using Mann-Whitney’s U test. Statistical significance was set at *p*<0.05. Analyses were performed using the EPI Info 7 program.

### Ethical considerations

The research project was submitted to the Research Ethics Committee of the HRMS and the Research Ethics Committee of the UFMS, with approval Nº 3.355.036.

## RESULTS

From 2017 to 2019, 154 patients were included in the study. Of these, 74.68% were male, aged 38–59 years (median 48 years). Most patients were non-white (79.87%) and lived in Campo Grande (74.03%). Resistance to certain drugs in the TB treatment regimen was observed in six (10.91%) patients (Table 1). Three patients were resistant to isoniazid, two were resistant to rifampicin, and one was resistant to streptomycin. Those who were resistant to rifampicin died.

**Table 1:** Demographics and clinical characteristics of the participants

Variables	N (%) or median [IQR]
Male	115 (74.68)
Age (in years)	48 [38–59]
Ethnicity	
White	31 (20.13)
Non-white	123 (79.87)
Education <9 years	78 (50.65)
Resident in Campo Grande – MS	114 (74.03)
PTB without extrapulmonary manifestation	123/141 (87.23)
PTB associated or not with extrapulmonary manifestation	141 (91.56)
Exclusive extrapulmonary TB	13 (8.44)
Laboratory confirmed TB	99 (64.29)
Smear	70 (45.45)
Culture	51 (33.12)
RMT GeneXpert *M. tb*/RIF	72 (46.75)
Main extrapulmonary sites	
Pleural	23 (14.94)
Ganglion	21 (13.64)
Meningeal	6 (3.90)
Vertebral	4 (2.60)
Peritoneal	4 (2.60)
Pericardial	3 (1.95)
PLHIV	13 (8.44)
CD4 +	62,5 [31–131]
Viral load	121.890,5 [49,894.5–145,832.5]
Resistance to TB treatment	6/55 (10.91)
TB treatment dropout history	9 (5.84)
History of deprivation of liberty	3 (1.95)
Other comorbidities	
DM	24 (15.58)
SAH	29 (18.83)
COPD	8 (5.19)
Malignant neoplastic disease	4 (2.60)
Hospitalization time	12 [5–21]
Treatment with RIPE	135/154 (87.66)
ICU indication	44 (28.57)
PLHIV in ICU	6/44 (13.63)
PTB without extrapulmonary manifestation in ICU	33/44 (75)
PTB associated or not with extrapulmonary manifestation in ICU	41/44 (93.18)
Exclusive extrapulmonary TB in ICU	3/44 (6.81)
Treatment with RIPE in ICU	40/44 (90.90)
Death	51 (33.12)
By septic shock	36/51 (70.59) *
By respiratory failure	18/51 (35.29) *
By bacterial infection	10/51 (19.61) *
By kidney failure	1/51 (1.96)

IQR = interquartile range; N = number; MS = Mato Grosso do Sul State; TB = tuberculosis; TBP = pulmonary tuberculosis; RMT = rapid molecular test; *M. tb*/RIF = *Mycobacterium tuberculosis*/rifampicin; PLHIV = person living with HIV; HIV = human immunodeficiency virus; DM = diabetes mellitus; SAH = systemic arterial hypertension; COPD = chronic obstructive pulmonary disease; ICU = intensive care unit; RIPE = rifampicin, isoniazid, pyrazinamide, ethambutol; *More than one cause of death was considered.

The case fatality rate among the patients was 33.12%, and the main cause of death was septic shock (70.59%) (Table 1).

Pulmonary tuberculosis (PTB) was the main clinical presentation (91.56%), and was exclusively pulmonary in 123 (87.23 %) patients. Laboratory confirmation of TB was performed in 99 (64.29%) patients. Among extrapulmonary manifestations, the pleural and lymph node sites were the most frequent (14.94% and 13.64%, respectively). The main treatment used was rifampicin, isoniazid, pyrazinamide, and ethambutol (RIPE) (87.66%) (Table 1).

A total of 13 (8.44%) patients were PLHIV, and in eight (61.5%) of these patients, TB manifested before the diagnosis of acquired immunodeficiency syndrome (Table 1).

The main comorbidities were systemic arterial hypertension (SAH) and diabetes mellitus (DM), which were present in 18.83% and 15.58% of patients, respectively. The median length of hospitalization was 12 days (Table 1).

Among patients admitted to the intensive care unit (ICU), only 13.63% were PLHIV. PTB was the main clinical presentation, with or without extrapulmonary manifestation (93.18%). The main treatment for patients in the ICU was RIPE (90.90%), administered either orally or through a nasoenteral tube (Table 1).

The patients who died were older, with a median age of 54 years (interquartile range [IQR], 45–61 years). Apart from age, there was no association between other epidemiological variables and death (Table 2).

**Table 2:** Epidemiological variables of the participants based on death during the hospitalization period

Characteristic	Death Yes N (%) or Med [IQR] N=51	Death No N (%) Med [IQR] N=103	*p*-value
Male	40 (78.43)	75 (72.82)	0.577
Age	54 [45–61]	44 [34–56]	0.001
Ethnicity			
White	10 (19.61)	21 (20.39)	1.000
Non-white	41 (80.39)	82 (79.61)	
Years of study			
<=9	28 (54.90)	50 (48.54)	0.567
>9	23 (45.10)	53 (51.46)	
Smoking	24 (48.00)	39 (38.24)	0.331
Alcoholism	21 (42.00)	27 (26.21)	0.074
Use of illicit drugs	4 (8.00)	13 (12.62)	0.562
Residents in Campo Grande – MS	33 (64.71)	81 (78.64)	0.096
PLHIV	6 (11.76)	7 (6.80)	0.461
Deprivation of liberty	1 (1.96)	2 (1.94)	1.000
History of treated TB	7 (13.73)	6 (5.83)	0.176
Time of onset of symptoms	14 [5–60]	15 [7–60]	0.737
TB treatment dropout history	5 (9.80)	4 (3.88)	0.267

MS = Mato Grosso do Sul State; IQR = interquartile range; N = number; PLHIV = persons living with HIV; HIV = human immunodeficiency virus; TB = tuberculosis; categorical variables were compared using the chi-square test or Fisher’s exact test; numerical variables were compared using Mann-Whitney’s U test; statistical significance was set at *p*<0.05.

Higher mortality rates were observed in patients with central nervous system (CNS) involvement, those admitted to the ICU, and those requiring mechanical ventilation (MV) or vasoactive drugs (VAD) (Table 3).

**Table 3:** Clinical variables of the participants based on death during the hospitalization period

Parameter	Death Yes N (%) or Med [IQR] N=51	Death No N (%) Med [IQR] N=103	*p*-value
Exclusive PTB	42 (91.30)	81 (85.26)	0.460
PTB and extrapulmonary TB	46 (90.20)	95 (92.23)	0.904
Exclusive extrapulmonary TB	5 (9.80)	8 (7.77)	0.905
TB in CNS	5 (9.80)	1 (0.97)	0.026
Confirmed TB	31 (60.78)	68 (66.02)	0.646
Clinically diagnosed TB	20 (39.22)	35 (33.98)	
DM	8 (15.69)	16 (15.53)	1.000
SAH	13 (25.49)	16 (15.53)	0.205
COPD	3 (5.88)	5 (4.85)	1.000
Hematologic disease	2 (3.92)	2 (1.94)	0.850
Drug-resistant TB	2 (9.52)	4 (11.76)	1.000
Hospitalization days	15 [5–22]	11.5 [5–20]	0.530
ICU admission	39 (76.47)	5 (4.85)	< 0.001
ICU stay days	12 [5–18]	4 [2–12]	0.122
Treatment with RIPE in ICU	35/40 (89.74)	4/4 (10.26)	0.61
PLHIV in ICU	5/6 (12.82)	34/38 (87.18)	0.54
Use of MV	42 (82.35)	5 (4.85)	< 0.001
Days on MV	11 [3–18]	5 [2–9]	0.103
Vasoactive drug use	39 (76.47)	3 (2.91)	< 0.001
Days on vasoactive drug	7 [3–15]	2 [2–7]	0.038
Apache II	27 [22–30]	25 [25–25]	0.580

IQR = interquartile range; N = number; TB = tuberculosis; PTB = pulmonary tuberculosis; CNS = central nervous system; DM = diabetes mellitus; SAH = systemic arterial hypertension; COPD = chronic obstructive pulmonary disease; ICU = intensive care unit; MV = mechanical ventilation; PLHIV = patient living with HIV; RIPE = rifampicin, isoniazid, pyrazinamide, ethambutol.

Among the main symptoms, a reduced level of consciousness was the most frequent in patients who died (Table 4).

**Table 4:** Main symptoms presented by patients diagnosed with tuberculosis based on death during hospitalization

Symptom	Death Yes N (%)	Death No N (%)	*p*-value
Fever	32 (62.75)	76 (73.79)	0.222
Cough	29 (56.86)	80 (77.67)	0.013
Expectoration	24 (47.06)	52 (50.49)	0.819
Dyspnea	44 (86.27)	77 (74.76)	0.153
Chest pain	31 (60.78)	54 (52.43)	0.418
Hemoptysis	10 (19.61)	15 (14.56)	0.571
Night sweats	17 (33.33)	45 (43.69)	0.290
Weight loss >10%	31 (62.00)	49 (48.04)	0.148
Asthenia	40 (78.43)	71 (68.93)	0.296
Headache	12 (23.53)	13 (12.62)	0.135

N = number; categorical variables were compared using the chi-squared test or Fisher’s exact test; statistical significance was set at *p*<0.05.

## DISCUSSION

This study was conducted in a tertiary hospital in Mato Grosso do Sul, Brazil, which serves critically ill patients. The study findings highlight a significant mortality rate among hospitalized patients with TB, underscoring the critical challenges in managing severe TB cases. The 33.12% mortality was influenced by older age and CNS involvement. Moreover, mortality was associated with ICU admission, MV, and the use of VAD, reflecting the complex interplay between TB and comorbid conditions, as well as disease severity in critically ill patients.

Most study patients were male, with a median age of 48 years, mainly residing in Campo Grande city, Mato Grosso do Sul State. These data are consistent with the 2024 Brazilian Epidemiological Bulletin of Tuberculosis, which reported that 69.2% of tuberculosis cases were notified in males; however, 33.8% were aged from 20 to 34 years^6^.

The patients who died were older, with a median age of 54 years (IQR, 45–61 years). In a South Korean study, 68% of patients were male, and 78.60% of deaths were observed in patients over 65 years of age. In the same study, multivariate analysis showed that smoking, history of TB, and coughing were significantly associated with TB mortality^12^.

Although PTB was the main clinical presentation, and the fact that TB was exclusively pulmonary in 87.23% of cases, this presentation was not associated with death. Regarding lifestyle habits such as smoking and drug use, no significant differences were identified in TB deaths. We did not observe a significant difference between the groups concerning alcohol use. However, our results showed a *p*-value of 0.076; hence, the lack of significance should be cautiously interpreted. In a 2018 cohort study, alcoholism was one of the most prevalent conditions among patients with TB who died^13^.

In this study, HIV infection was not associated with mortality. However, PLHIV are at a high risk of developing active TB and are associated with unfavorable outcomes. Several strategies have been introduced to reduce deaths^14^. Although some low- or middle-income countries have achieved at least a 75% mortality reduction, most nations are not on track to meet this goal, continuing to record increasing deaths^15^.

The duration of hospitalization ranged from five to 21 days, with a median of 12 days. In a study that evaluated the length of hospital stay for TB, the median was 14 days (IQR, 6–22), but this duration increased with TB severity and patients’ older age^16^.

The main comorbidities observed in patients with TB were SAH, DM, and chronic obstructive pulmonary disease (COPD). It is estimated that patients with DM are four times more likely to develop PTB, with a prevalence of approximately 76% in patients with TB^17^. Other studies have demonstrated the association of DM and COPD with TB, which were the second and third most frequent comorbidities in our study, respectively^18-20^.

Although our data showed no association between the observed comorbidities and mortality, COPD was one of the most frequent comorbidities in patients who died of TB in a 2018 cohort study. In the same study, SAH was not mentioned, and DM showed no difference in the control group^13^.

CNS TB was the third most frequent extrapulmonary manifestation in the study patients (3.9%) and was also prevalent in patients who died (83.33%), which is an important factor associated with death in the aforementioned cohort, presenting a higher mortality rate than PTB and other extrapulmonary TB^13^.

Among the main symptoms, a reduced level of consciousness was more frequent in patients who died. This may be related to TB cases in the CNS, critically ill patients admitted to the ICU, and the use of MV and VAD, all of which were more prevalent in patients who died. Cardiorespiratory failure has been reported as a direct cause of early death from TB^21-23^. In other studies, acute respiratory failure, septic shock, and multiple organ dysfunction were the most frequent reasons for ICU admission in patients with active TB^24-26^.

Bacterial coinfection was the third leading cause of death in our study and is also a common cause of critical illness in patients with active TB^27^. In addition, the frequency of deaths in patients with confirmed TB requiring ICU admission was high, reaching 68.70%^26,28,29^. In our study, there was an overall frequency of death of 33.12% and 88.64% in patients with ICU indications.

MV and the presence of shock are factors with a high predictive value for mortality, and were the primary determinants of death^30^.

Generally, the hospital mortality rate ranges from 12.3% to 42.5% in several countries; these differences may be related to the populations studied, which can be influenced by ethnicity and comorbidities. TB mortality rates in long-term studies, including outpatients and hospitalized patients, are notably lower. The overall long-term mortality associated with TB is 0.14% in Poland and 6% in Saudi Arabia. This discrepancy indicates that the highest mortality rate in patients with TB occurs during the initial phase of the disease or during hospitalization^10,31^, as was observed in our study.

The high frequency of deaths observed in our study was associated with clinical characteristics indicative of severe disease, respiratory complications, and the need for MV and VAD. Therefore, it is crucial to analyze potential causes of these unfavorable outcomes. One significant factor was that the most essential anti-TB medication was orally administered. Furthermore, the scarcity of injectable drugs combined with impaired absorption of oral medications in patients using VAD likely contributes to mortality^29^.

In Brazil, in 2022, the Mato Grosso do Sul State was among the four states with the highest TB mortality rates, along with Amazonas, Rio de Janeiro, and Pará States. Unfortunately, the COVID-19 pandemic has significantly contributed to delays in the diagnosis and treatment of active TB^6^. Additionally, our findings highlight the need for more effective management strategies for critically ill patients with TB.

The main limitations of the study were its retrospective design, and the use of secondary data obtained from a database and electronic medical records, which may not represent all detailed clinical information.

## CONCLUSION

In conclusion, this study highlights the high mortality rate among hospitalized patients with TB, particularly in those with older age, CNS involvement, ICU admission, MV use, and VAD use. These findings underscore the urgent need for improved clinical management strategies, including early diagnosis, timely and appropriate treatment interventions, and enhanced supportive care. Addressing the specific challenges of managing critically ill patients with TB in high-complexity hospital settings is essential to reduce mortality and improve patient outcomes.
